# 应用二代流式细胞术和二代测序评估多发性骨髓瘤微小残留病比较

**DOI:** 10.3760/cma.j.issn.0253-2727.2023.04.011

**Published:** 2023-04

**Authors:** 晴晴 王, 利 姚, 明清 朱, 灵芝 颜, 松 金, 京晶 商, 晓兰 施, 英颖 翟, 霜 颜, 卫芹 姚, 红英 尤, 德沛 吴, 琤琤 傅

**Affiliations:** 苏州大学附属第一医院，江苏省血液研究所，国家血液系统疾病临床医学研究中心，国家卫生健康委员会血栓及止血重点研究室，苏州 215000 Jiangsu Institute of Hematology, National Clinical Research Center for Hematologic Diseases, NHC Key Laboratory of Thrombosis and Hemostasis, the First Affiliated Hospital of Soochow University, Suzhou 215000, China

多发性骨髓瘤（MM）是一种以克隆性浆细胞恶性增殖为特征的疾病，发病率在血液系统恶性肿瘤中排名第二位，目前仍无法治愈[1]。近年来，随着免疫调节药物、蛋白酶体抑制剂、CD38单抗等新药及自体造血干细胞移植（ASCT）技术的应用，MM的完全缓解（CR）率不断提高，但几乎所有病例仍会复发[2]–[5]。2016年，国际骨髓瘤工作组（IMWG）将微小残留病（MRD）列入疗效评估[6]。MRD是治疗后或治疗期间持续存在少数恶性细胞的状态，被认为是复发的根源，故亟需更敏感的检测技术评估深层缓解程度。目前MRD检测技术主要包括二代流式细胞术（NGF）、二代测序（NGS）、等位基因特异性寡核苷酸聚合酶链反应技术（ASO-PCR）、体液活检及影像学技术等。这些技术各有其优点和局限性，尚无统一的检测方法与评价标准。

本研究中我们应用NGF及NGS方法动态监测同一患者的骨髓MRD情况，统计分析最低检测线（limit of detection，LOD）及肿瘤负荷（tumor load，TL），在保证两种方法LOD达到1.0×10^−5^的基础上定义MRD阴性，并进行MRD结果的逐步统计分析，证实其对深度缓解的评估作用，分析比较两种方法的一致性及在检出率上的差异，同时探索MRD检测对患者生存的预测。

## 病例与方法

1. 研究对象：本中心的一项VRD登记性研究纳入苏州大学附属第一医院自2019年9月1日至2022年1月31日收治的82例初诊MM患者，在治疗过程中同时采用NGF及NGS方法检测患者骨髓MRD情况。MM诊断、分期标准及疗效评估参照2020年修订版中国多发性骨髓瘤诊治指南[7]。监测的时间点为诱导治疗后、移植/巩固治疗后，维持治疗期间每半年进行一次。

2. NGF：取MM患者的新鲜骨髓标本（24～48 h），选取骨髓涂片后的第一管骨髓液（3～5 ml）。抗体包括CD138-APC、CD38-APC750、CD45-KO、CD19-ECD、CD56-PC7、CD27-PB、CD81-APC700、CD117-PC5 8种膜抗体以及Kappa-FITC、Lambda-PE两种胞质抗体（Kappa-FITC和Lambda-PE购自丹麦Dako公司，膜抗体购自美国Beckman Coulter公司）[8]。

3. NGS：先确定初诊MM患者全骨髓细胞中肿瘤浆细胞免疫球蛋白克隆性重排（美国Invivoscribe公司的LymphoTrack检测试剂盒），包括IGH和（或）IGK基因克隆性重排的类型和序列。在患者后续的随访标本中，以诊断时检测到的克隆性重排序列作为其分子标志来进行MRD监测[9]。

4. 随访：随访时间截至2022年1月31日。中位随访时间为14（5～29）个月。通过查阅住院病历、门诊病历及电话进行随访。对于随访期间死亡的病例，通过病历记录和（或）与患者家属电话联系确认。总生存（OS）时间定义为自确诊之日至末次随访或死亡的时间。无进展生存（PFS）时间定义为自确诊之日至疾病发生进展或死亡的时间。

5. 统计学处理：采用SPSS 26.0软件及GraphPad Prism 9.0进行统计学分析。计数资料用例数（百分比）表示，计量资料用中位数（范围）表示。均数的比较采用成组两样本秩和检验和方差分析的双侧检验，率的比较采用Fisher确切概率法；两项数据的一致性检验采用Kappa系数，定量数据的相关性分析采用Pearson积矩相关系数。采用Kaplan-Meier法绘制生存曲线，Log-rank检验用于估计单个危险因素的生存差异，*P*<0.05为差异有统计学意义。

## 结果

1. 一般资料：共有82例同时进行NGF-MRD及NGS-MRD检测的MM患者纳入最终分析，诱导治疗后疗效达到非常好的部分缓解（VGPR）以上者71例（86.59％），疗效达CR及严格意义的CR（sCR）患者44例（53.66％），移植或非移植患者完成VRD方案治疗（共63例）后疗效达VGPR以上者59例（93.65％），达CR+sCR者41例（65.08％）。

2. NGF及NGS方法检测的LOD值及TL：从样本采集到系统输出均影响MRD的测定，其结果高度依赖于样本的质量及浓度[10]–[13]。统计82例患者诱导治疗后用于NGF-MRD及NGS-MRD检测的细胞总数，分别统计两种检测方法的中位LOD值和中位TL（图1）。本研究中，将在1.0×10^−5^水平未检测到肿瘤细胞定义为NGF-MRD阴性或NGS-MRD阴性。去除3例未达标的NGS标本，分析在同一检测标准下检测到的MRD结果，42例NGF-MRD阳性，64例NGS-MRD阳性，应用NGS方法检测出的MRD阳性率较应用NGF方法更高（81.01％对51.22％）。其中，将MRD阴性标本的LOD值视为TL纳入研究，NGS较NGF能检测出更高的TL（中位TL：2.440×10^−3^对1.155×10^−5^，*P*<0.001）。

**图1:**
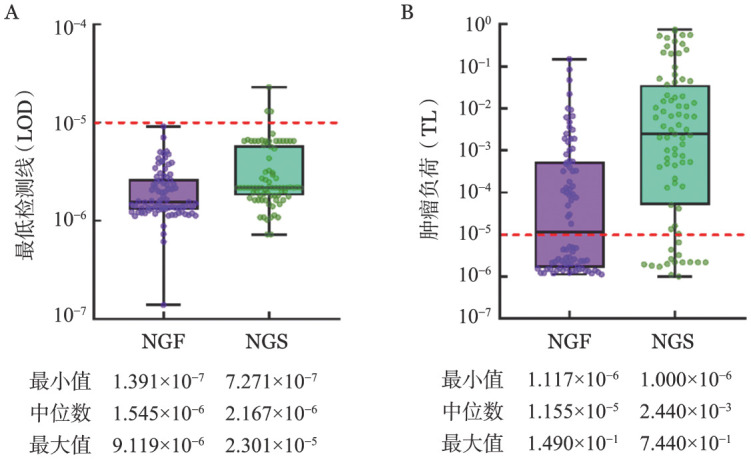
应用二代流式细胞术（NGF）及二代测序（NGS）分析82例多发性骨髓瘤患者微小残留病（MRD）最低检测线（A）及相应肿瘤负荷（B） 在应用NGS检测MRD时，按照每个细胞提取6.5 pg DNA计算

3. 不同疗效下的MRD分析：分析82例患者在传统IMWG疗效标准评估下的MRD状况，疗效在VGPR以下的患者无论应用NGF还是NGS检测，MRD结果均为阳性。8例（29.63％）疗效为VGPR的患者NGF-MRD为阴性，CR以上患者NGF-MRD阴性率则达到75％。疗效为VGPR的患者NGS-MRD阴性率为3.85％，CR以上的患者NGS-MRD阴性率为32.56％。当疗效达到VGPR以上再进行MRD检测似乎更为合理[13]–[16]。随着疗效的不断加深，MRD阳性率逐渐降低，中位TL逐渐减低，疗效为疾病进展（PD）的患者TL最高，而疗效达到CR的患者中位TL值最低（图2）。MRD不仅能精准地呈现不同疗效水平患者TL的差距（*P*＝0.002），同时也能在一定程度上说明同一疗效水平患者TL不同，总之，MRD反映更深层次的缓解。

**图2:**
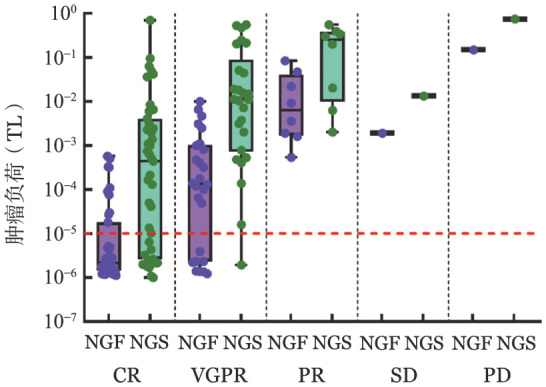
应用NGF及NGS分析最低检测线值达到1.0×10^−5^水平的79例多发性骨髓瘤患者不同疗效下的肿瘤负荷 NGF：二代流式细胞术；NGS：二代测序；CR：完全缓解；VGPR：非常好的部分缓解；PR：部分缓解；SD：疾病稳定；PD：疾病进展

将NGF-MRD与NGS-MRD结果进行配对统计学分析，就MRD的检出率而言，对于疗效为VGPR、CR及sCR的患者，NGS-MRD的阳性检出率较NGF-MRD更高（VGPR：96.15％对70.37％；CR及sCR：68.18％对25.00％）。同样，在TL的检测方面，NGS较NGF能检测到更高的TL，不同疗效的中位TL如下：部分缓解（PR）：2.505×10^−1^对6.400×10^−3^，*P*＝0.002；VGPR：1.120×10^−2^对1.350×10^−4^，*P*＝0.005；CR及sCR：4.410×10^−4^对2.153×10^−6^，*P*＝0.023，差异均有统计学意义。

4. 一致性：去除LOD值未达到1.0×10^−5^的3例患者，对疗效达到VGPR及以上的69例患者MRD结果进行配对分析，15例（21.74％）患者骨髓NGF-MRD与NGS-MRD均为阴性，29例（42.03％）患者两种检测方法均为阳性。在MRD定性方面，两种方法的一致率为63.77％，与国外结果（68.00％）相似[17]。两种检测方法结果不一致之处为：25例（36.23％）患者NGS检测MRD为阳性，但应用NGF检测为阴性，不存在NGF-MRD阳性而NGS-MRD阴性的情况。应用Fisher确切法比较两种方法是否具有一致性，结果表明*P*<0.001，两种方法结果一致，差异有统计学意义。但Kappa＝3.728，一致性较差。

除去LOD值未达到1.0×10^−5^的3例患者，对疗效达到VGPR以上且MRD阳性的54例患者进一步行配对分析比较两种方法TL的差异，相关系数*r*＝0.4371（54例，*P*＝0.001）（图3）。综上，两种方法在MRD结果定性及TL定量方面均有一定的相关性。

**图3:**
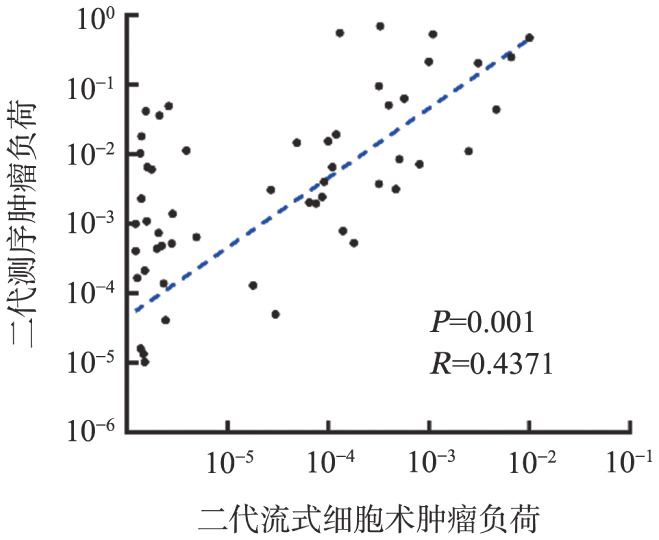
54例多发性骨髓瘤患者微小残留病定量结果的一致性分析

5. 生存分析：对79例LOD值达到1.0×10^−5^的患者进行生存分析，中位随访时间为14个月，共6例患者进展，2例患者死亡，中位PFS时间及OS时间均未达到。根据诱导结束后的疗效将79例患者分为CR及sCR、VGPR和PR及以下3个组别，三组患者的中位PFS时间分别为未达到、未达到和17个月（*P*<0.001），中位OS时间均未达到（*P*＝0.535）（图4）。2例死亡患者中1例死于肺部感染，另1例死于疾病进展。

**图4:**
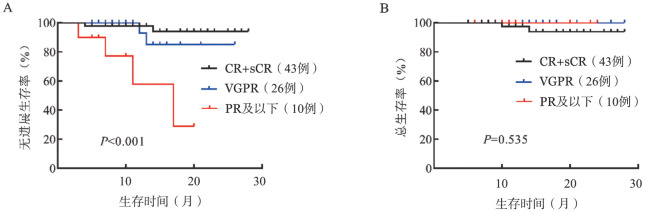
不同疗效组79例多发性骨髓瘤患者的无进展生存（A）及总生存（B）曲线 CR：完全缓解；sCR：严格意义的完全缓解；VGPR：非常好的部分缓解；PR：部分缓解

将疗效CR+sCR组和VGPR组单独进行比较，24个月PFS率分别为94.06％和85.12％，差异无统计学意义（*P*＝0.413）。根据MRD结果对LOD值达到1.0×10^−5^且疗效达到VGPR及以上的69例患者进行分组，中位随访时间15个月，4例进展，2例死亡。所有MRD阴性患者均未进展或死亡。在应用NGF方法检测时，MRD阴性组的PFS时间较阳性组延长（*P*＝0.008），应用NGS检测时，MRD阳性组与阴性组PFS时间的差异无统计学意义（*P*＝0.234）（图5）。两种方法OS的差异均无统计学意义（*P*值均>0.05）。

**图5:**
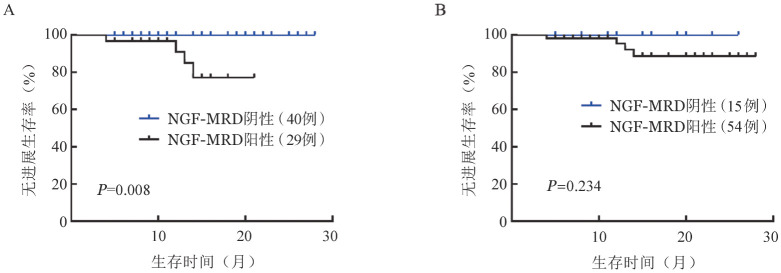
应用NGF（A）及NGS（B）比较MRD阴性组和阳性组的无进展生存情况 NGF：二代流式细胞术；NGS：二代测序；MRD：微小残留病

根据69例传统疗效达到VGPR及以上患者的MRD结果进行分组，共分为四组：NGF-MRD阴性且NGS-MRD阴性组（15例），NGF-MRD阴性且NGS-MRD阳性组（25例），NGF-MRD阳性且NGS-MRD阴性组（0例），NGF-MRD阳性且NGS-MRD阳性组（29例）。所有进展及死亡患者均为NGF-MRD阳性且NGS-MRD阳性。各组PFS的差异有统计学意义（*P*＝0.029），OS的差异无统计学意义（*P*＝0.162）。

## 讨论

MM治疗的CR率不断提高，经过治疗达到CR的患者预后仍然不同，即便之后提出了sCR的概念，仍不能很好地解释CR患者结局的差异性。2016年，国际骨髓瘤工作组（IMWG）将MRD列入疗效评估[6]。但由于利用不同的技术、研究设计、统计标准，在MM临床试验中评估和报告MRD的方式存在显著差异，这种差异为数据解释、后续研究的设计和定量荟萃分析带来了挑战。因此多个指南及共识推荐在进行MM-MRD的分析时，必须以标准化的方式定义、评估和报告，特别是LOD、MRD阴性的界定等。

本研究以1.0×10^−5^为LOD值，100％的NGF标本及96.34％的NGS标本达到此标准。随着疗效反应的加深，两种方法检测的MRD阳性率逐步下降。疗效为PD、疾病稳定、PR的患者NGS-MRD及NGF-MRD结果皆为阳性，说明我们在进行MRD的评估时要求疗效达到VGPR及以上。诱导治疗后应用NGF检测，疗效达到VGPR以上的患者MRD阳性率为42.25％。Kriegsmann等[17]的研究数据显示，应用NGF检测，疗效达到VGPR以上的MRD阳性患者占42.02％，Flores-Montero等[18]的研究数据为47.00％。应用NGS方法检测，疗效达到CR以上患者的MRD阳性率为67.44％。GRIFFIN临床研究结果表明，VRD组疗效达CR及以上的患者NGS-MRD阳性率为67.80％[19]。本研究结果同时也反映，随着疗效的不断加深，MRD不仅能精准地呈现不同疗效水平上的TL差距，同时也能在一定程度上说明同一疗效水平的患者TL可能不同。就MRD的检出率而言，NGS的MRD阳性检出率更高，在TL的检测方面，应用NGS可检测到更多的肿瘤细胞数量。将NGF-MRD与NGS-MRD结果进行配对分析，显示两种方法在MRD结果定性及TL定量方面有一定的相关性。42.03％的患者两种检测方法均为阳性。21.74％均为阴性，总的一致率为63.77％，与国外结果相似[17],[20]。Medina等[21]的研究认为两者的一致性可达到85.8％，但该研究NGF的LOD值达2×10^−6^水平，NGS的LOD值为10^−5^水平。在CASSIOPEIA试验中，两种方法的总体一致率为83.5％，但该研究并未标注LOD值[22]。在生存分析中，NGF-MRD阳性预示更短的PFS时间、早期复发。NGF和NGS检测MRD阴性预测2年PFS率100％。由于本研究随访时间较短，仅对诱导治疗后MRD结果进行分析，后期我们将对这些患者继续随访，探讨不同监测时间点的MRD阳性率及MRD水平对患者OS的影响。

NGF和NGS为两种常用的检测骨髓中MRD的技术，两种技术各有其优点及局限性。NGF检测MRD所需的骨髓标本量较多，且必须为新鲜标本，需在24～48 h内评估，但其有适用性广、检测迅速、简便等优点。NGS检测MRD所需的骨髓标本量少，可检测保存时间较长的标本，有利于回顾性分析，但该技术仅适用于90％的患者，且需要与基线标本进行对比[23]–[25]，具有检测周期长、检测费用高等局限性，具备该技术的实验室也较少[26]–[27]。由于NGS较NGF有更高的敏感性，本研究将继续延长随访时间，观察MRD检测结果对PFS的影响。

由于MRD检测在MM的疗效评估、预后判断、复发预测、治疗指导等方面具有重要意义，为提高检测结果的稳定性、可比性及可信度，亟需制定检测技术的统一标准。
